# Supplementary figures and images for: Sand flies and Toscana virus: Intra-vector infection dynamics and impact on Phlebotomus perniciosus life-history traits
Source: PLoS Negl Trop Dis. 2024 Sep 25;18(9):e0012509. doi: 10.1371/journal.pntd.0012509 (PMC11458028; doi:10.1371/journal.pntd.0012509)

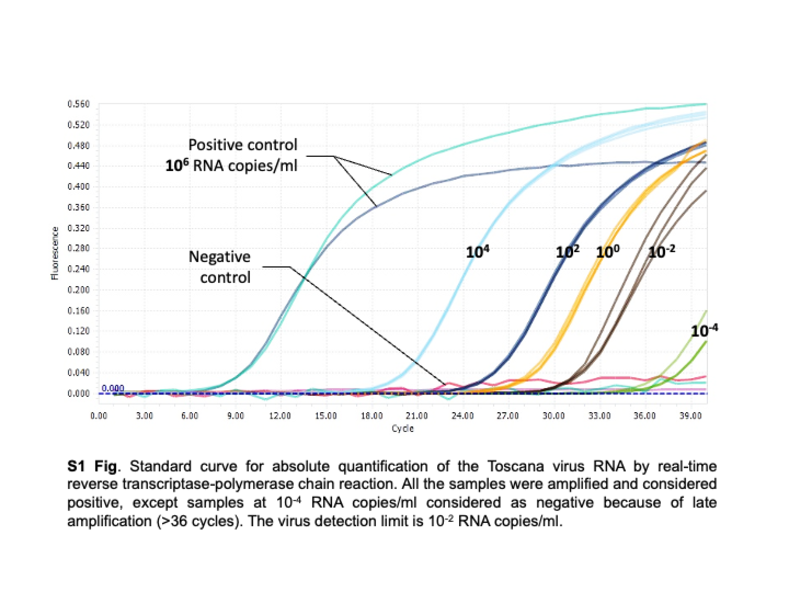

Supplement: S1 Fig — All the samples were amplified and considered positive, except samples at 10−4 RNA copies/ml considered as negative because of late amplification (>36 cycles). The virus detection limit is 10−2 RNA copies/ml. (TIF) [file pntd.0012509.s001.tif]

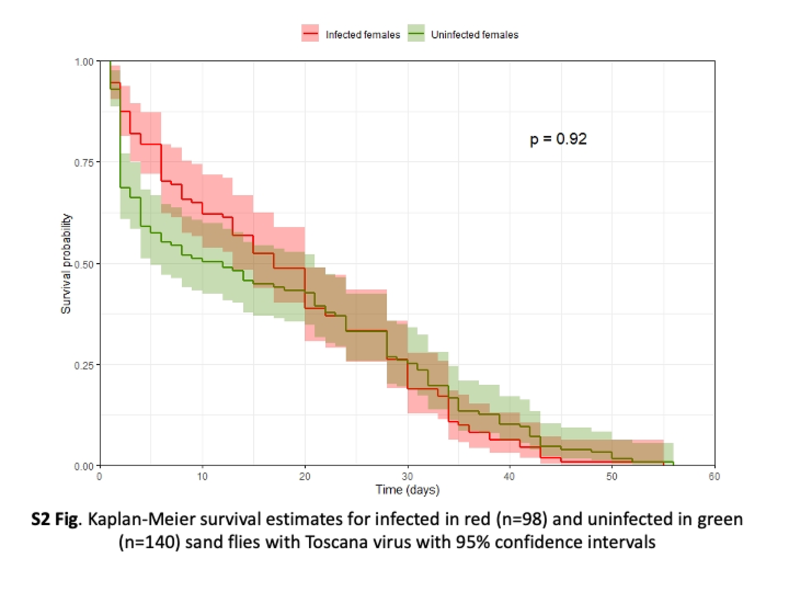

Supplement: S2 Fig — (TIF) [file pntd.0012509.s002.tif]

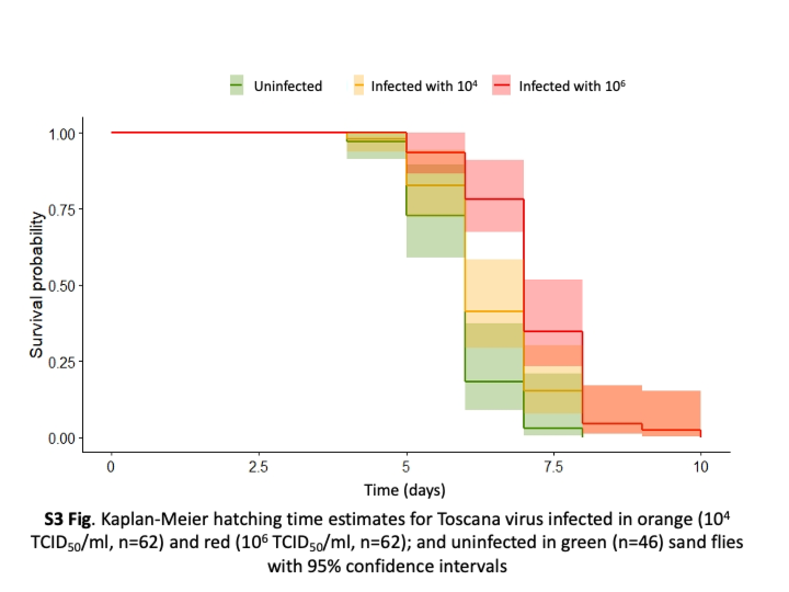

Supplement: S3 Fig — (TIF) [file pntd.0012509.s003.tif]
