# Supplementary material for: Sand flies and Toscana virus: Intra-vector infection dynamics and impact on Phlebotomus perniciosus life-history traits
Source: PLoS Negl Trop Dis. 2024 Sep 25;18(9):e0012509. doi: 10.1371/journal.pntd.0012509 (PMC11458028; doi:10.1371/journal.pntd.0012509)
Supplement: Attachment — Submitted filename: Comments-Reviewer.docx [file pntd.0012509.s006.docx]

The manuscript entitled **“**Sand flies and Toscana virus: intra-vector infection dynamics and impact on *Phlebotomus perniciosus* life-history traits” describes an interesting study. The results of this study are of major eco-epidemiological importance and should lead to a better to a better understanding of the impact of phleboviral infection the vector biology of the sand fly vector. The manuscript should be published after taking into consideration the following comments:

**Abstract:**

Line 30: Add other countries from Southern Europe as Spain and Portugal

**Introduction**

Lines 73-74: Add references

Line 76: Change to “is related to competent sand fly vectors”

Line 79: Elaborate more this sentence because it is confusing. According to the ref (8), there is no maintenance of virus.

Line 83: Please define “infection dynamics”

Line 85: Ref (6) is not appropriate

Line 86: Please elaborate more the infection of males with TOSV in nature.

Line 93: Add mosquito-borne arboviruses

Line 95: Add a sentence stating that most research concerned mosquito-borne arboviruses compared to studies on sandfly-borne arboviruses.

Line 99: This reference do not concern TOSV. Delete "including TOSV"

Line 101: This statment concerned mosquitoes. Thus, the authors should state "arbovirus infection in mosquitoes"

Line 107: What does it mean infection dynamics in Ph. perniciosus? The vector competence of Ph. perniciosus is already confirmed.

Line 110: The authors studied the impact of TOSV infection on the fecundity and survival and NOT on the transmission dynamics.

**Methods**

Line 118: From which generation, the authors started the colonization?

Line 119: Add the paper of Maroli et al. 2007, Chelbi and Zhioua, 2007, 2019

Line 119: Add a published reference and not submitted (Ref 28 is not acceptable).

Line 132: Please provide the origin of the strains and from which sandfly species it was isolated. Also, add the Gen Bank Accession Number of the complete sequence of the TOSV strain if it has been published in the GenBank database.

Line 138: Please specify at which passage of viral culture, the TCID50/ml was calculated as well as the name of the method (ref) used in calculation.

Line 149: Add the reference of Maroli et al. 2007.

Line 150-151: The authors should report which passages of viral cultures related to these three concentrations

Line 152: Are you using the same generation of Ph. perniciosus? Did the authors examine viral infection in Ph. perniciosus prior to the experiment?

Line 168: What type of measures taken to prevent cross contamination between parts. Salivary glands is the most important organ to be examined after blood digestion.

Line 169: Why including wings and legs? The authors should differentiate between engorged females and females that digested blood. This have to be discussed. Most importantly is to test eggs, larvae, and emerging adults males and females for viral infection.

Line 172: Please explain the rationale of using only dose 3.

Line 181: Add the % of hatching.

Line 184: “adding sand” Please add the reference of Chelbi and Zhioua, 2007.

Line 190: The cannibalism occurs only when food for larvae is enough. This is not an acceptable reasons. The authors should follow up the life cycle until emergence of adults to be examined for transovarial transmission. The authors should let larvae accomplish their cycle and test the presence of TOSv in eggs, larvae, and emerging adult.

Line 195: This is an unacceptable reference (submitted). The authors do not have a long history of sand fly colonization. Reducing the T°C will make the oviposition period longer. Engoged females that do not have access to an egg-laying post will cause a significant stress factors. This must be discussed.

Line 197 : Specify these conditions

Line 202 : It could be interesting to use a lower dose close to natural transmission

Line 233: The authors should define in MM the term infection dynamics. It means to quatify the viral infection in the gut and in hoemocel, then in the salivary galnds. It is a continious process.

Line 234 : Define in MM systemic infection

**Results**:

Line 247: This daily mortality is high. Could you explain the reasons?

Line 282: Delete “the estimation from 0 to 5”

Line 295: 9280 ???

Line 305-310: It is very important here to clearly clarify the sugar cotton analysis result for the three doses. Did the three batches of females infected with doses 1, 2 and 3 release the virus during sugar feeding.

**Discussion**:

Line 360: Please note that microbiota has a great impact of the vector competence.

Line 366: How you explain the presence of TOSV in wings and legs?

Line 368: Ph. perniciosus as the main vector of TOSV in nature is already confirmed. Thus, the authors only quantified the virus during its replication and dissemination.

Line 380: The authors should always use the term mosquito-borne arboviruses. It is misleading to use the term arboviruses in the discussion.

Line 387: Please note that the infection related to Ref (12) is through intra-thoracic injection and therefore, you cannot make comparison.

Line 391: Please provide other convincing explanation beside the use of different strain.

Line 392: Not sure, the author should check the dynamics of viral infection in the vector. First it decreases followed by a replication and dessimination.

Line 393 : Ref (47) is not approriate.

Line 405: The authors are assuming that human is a reservoir host for TOSV. This is a miss leading statement and should deleted.

Line 408 : “female sand flies die before taking a second blood meal”. This is totally wrong. Ref 28 should be replaced. Again, the authors do not have good experience in sand fly biology.

Line 424: The authors should demonstrate infection during sugar co-feeding.

Line 427: The authors emphasize on the viral transmission through sugar feeding and making assumption with similarity for blood feeding viral transmission

**Most important concerns in the discussion**:

The authors should compare and discuss the difference between intrathoracic inoculation and membrane feeding. Sand flies are refractory to oral infection. Phlebovirus are maintained in nature through transovarial transmission. Please refer to Tesh and Modi, Am J Trop Med and Hyg 1984 33: 1007-1016.

In addition, the authors should provide convincing proof that wigs and legs were not infect through sugar feeding.

Why the authors exclude dogs from the study? This is an important missing data.

48 hrs after blood feeding, engorged sandflies were stored in 70% alcohol for further DNA extraction and qPCR for *L. donovani*. The normal cycle of *L. donovani* within the sandfly takes 8-10 days to reach stationary phase. Why the authors did not allow for a full cycle accomplishment of *L. donovani* within the sandfly.

**Results**

Since xenodiagnosis was repeated throughout the year, the authors should report results at each time.

**Discussion**

The authors should report studies concerning xenodiagnoses performed on humans.

Taking into account that dogs are considered as reservoir hosts for *L. donovani* (Jumbulingam et al., 2017), the authors should discuss the rationale of not testing dogs.
