# Supplementary material for: Sand flies and Toscana virus: Intra-vector infection dynamics and impact on Phlebotomus perniciosus life-history traits
Source: PLoS Negl Trop Dis. 2024 Sep 25;18(9):e0012509. doi: 10.1371/journal.pntd.0012509 (PMC11458028; doi:10.1371/journal.pntd.0012509)
Supplement: Attachment — Submitted filename: Answer letter_29-07-24.docx [file pntd.0012509.s007.docx]

**Manuscript** - PNTD-D-24-00437

**Title:** Sand flies and Toscana virus: intra-vector infection dynamics and impact on *Phlebotomus perniciosus* life-history traits

**Corresponding author:** Lison LAROCHE [lisonlaroche14@gmail.com](mailto:lisonlaroche14@gmail.com)

Dear Editorial-Board Member,

Our paper entitled “**Sand flies and Toscana virus: intra-vector infection dynamics and impact on *Phlebotomus perniciosus* life-history traits**” has been revised according to Editorial-board and Reviewer comments. Changes were included in the manuscript with the track changes mode. Please find below a detailed list of responses to the reviewer comments and a description of the changes made to the manuscript.

Yours sincerely,

Lison LAROCHE

**Reviewer #1**

Methods

The article Sand flies and Toscana virus: intra-vector infection dynamics and impact on Phlebotomus perniciosus life-history traits investigated the intra-vector TOSV infection dynamics in Ph. perniciosus, as well as its impact on the vector's life history traits. As reported by aithors limited research has been conducted on transmission dynamics and the vectorial competence and capacity of the principal TOSV vector, Phlebotomus perniciosus.

The objectives are clear and the hypothesis stated teastable and the study design appropriate

This manuscript is well written and results presented clearly with only minor formatting/typos that need to be addressed prior to publication

Answer: We thank the reviewer for these comments, which we have taken into account to improve the quality of the manuscript.

lines 78-79: please specify species

Answer: As requested by the reviewer, we have added line 101 examples of sand fly species likely to contribute to the virus maintenance and transmission.

73-79: please give more details on SF distribution in the mediterranean area especially for Ph. perniciosus

Answer: As requested by the reviewer, we have added details on sand fly distribution line 98.

93-103 This paragraph is very broad and general. I suggest summarising it or cutting it.

Answer: As requested by the reviewer, the paragraph has been summarised.

Results

117: please explain why you choose Ph. Perniciosus

Answer: As requested by the reviewer, we explained in this part lines 163-164 why we chose this species for our study.

250: Plead explain better in Table 1 that number represent Phlebotomus perniciosus dissected

Answer: As requested by the reviewer, we modified the title of table 1 line 314.

Figure 3: Add information on the number of doses to the x axis ie: Time post infection (days) (two doses).

Answer: As requested by the reviewer, we have added this information in the Figure 3 line 347.

Conclusions

Conclusions are well supported by data presented but discussion needs of a specific paragraph on the limitations of the study. Public health relevance is well adressed

Answer: We thank the reviewer for this comment and have improved the conclusions by adding more information about the limitations of the study at the end of the discussion.

Summary and General Comments

This manuscript is well written and results presented clearly.

Answer: We thank the reviewer for his/her general comment and the help provided to improve our manuscript.

**Reviewer #2**

The manuscript entitled **“**Sand flies and Toscana virus: intra-vector infection dynamics and impact on *Phlebotomus perniciosus* life-history traits” describes an interesting study. The results of this study are of major eco-epidemiological importance and should lead to a better to a better understanding of the impact of phleboviral infection the vector biology of the sand fly vector. The manuscript should be published after taking into consideration the following comments:

Answer: We thank the reviewer for these comments, which we have taken into account to improve the quality of the manuscript.

Abstract:

Line 30: Add other countries from Southern Europe as Spain and Portugal

Answer: As requested by the reviewer, we have added this information in the abstract line 34.

Introduction

Lines 73-74: Add references

Answer: As requested by the reviewer, two references were added line 95.

Line 76: Change to “is related to competent sand fly vectors”

Answer: As requested by the reviewer, we changed the sentence line 97.

Line 79: Elaborate more this sentence because it is confusing. According to the ref (8), there is no maintenance of virus.

Answer: As requested by the reviewer, we elaborate and corrected this sentence line 100: “However, other sand fly species may also contribute to the virus transmission, such as *Phlebotomus tobbi*, *Phlebotomus longicuspis* and *Sergentomyia minuta* (8)”. However, we don’t understand the last comment of the reviewer, because the reference 8 refers to current knowledge of entomology, epidemiology and virology supporting the possible existence of additional phlebotomine species in TOSV maintenance, but this article does not say that there is no maintenance of the virus. Moreover, reference 8 is an article written by some co-authors of the present manuscript.

Line 83: Please define “infection dynamics”

Answer: As requested by the reviewer, we completed the sentence as following lines 106-107: “However, to date, limited information is available on natural cycle and transmission of TOSV particularly with regard to the phlebovirus infection dynamics (the progression of the virus within a host organism over time) in the vectors or in animals or humans”.

Line 85: Ref (6) is not appropriate

Answer: As requested by the reviewer, we changed the reference line 109.

Line 86: Please elaborate more the infection of males with TOSV in nature.

Answer: As requested by the reviewer, we developed the infection of males with TOSV as following lines 117-120: “Additionally, TOSV has been isolated in Italy and Morocco (Verani *et al.* 1980; Es-Sette *et al.* 2012) from naturally infected males of *Ph perniciosus*, however males are not haematophagous and these results has been interpreted as evidence of transovarial transmission under natural conditions”.

*Verani P. Lopes M. C. Nicoletti L. Balducci M.. 1980. Studies on phlebotomus-transmitted viruses in Italy. Isolation and characterization of a sandfly fever Naples-like virus, pp. 195–201. In Vesenjak–Hirjan J. Porterfield J. S. Arslanagic E. (eds.), Arboviruses in the Mediterranean Countries. Gustav Fischer Verlag, Stuttgart, Germany.*

*N. Es-Sette, J. Nourlil, S. Hamdi, F. Mellouki, M. Lemrani, First Detection of Toscana Virus RNA From Sand Flies in the Genus Phlebotomus (Diptera: Phlebotomidae) Naturally Infected in Morocco, Journal of Medical Entomology, Volume 49, Issue 6, 1 December 2012, Pages 1507–1509, https://doi.org/10.1603/ME12042*

Line 93: Add mosquito-borne arboviruses

Answer: We consider preferable to use here the word arbovirus, as it also includes pathogens transmitted by other vectors than mosquitoes, such as ticks, which are responsible for an increasing number of cases.

Line 95: Add a sentence stating that most research concerned mosquito-borne arboviruses compared to studies on sandfly-borne arboviruses.

Answer: As requested by the reviewer, we added a sentence on the importance of mosquito-borne arboviruses line 129-131: “Most of this research has concerned mosquito-borne viruses compared to studies on sandfly-borne viruses, reflecting the historically higher impact of mosquito-transmitted diseases on public health”.

Line 99: This reference do not concern TOSV. Delete "including TOSV"

Answer: As requested by the reviewer, we modified the sentence line 134.

Line 101: This statment concerned mosquitoes. Thus, the authors should state "arbovirus infection in mosquitoes"

Answer: As requested by the reviewer, we have corrected the above-mentioned parts of our manuscript line 135-136.

Line 107: What does it mean infection dynamics in Ph. perniciosus? The vector competence of Ph. perniciosus is already confirmed.

Answer: According to the authors, no study has been published in a scientific journal on TOSV experimental infection dynamics (progression of the virus within an organism) within the vector *Ph. perniciosus*. The vector competence of *Ph. perniciosus* has been confirmed only after field collections in endemic areas. It is therefore important to study the kinetics of virus development in the vector and to show that *Ph. perniciosus* can be infected by TOSV through experimental infections to better understand how the transmission works in nature.

Line 110: The authors studied the impact of TOSV infection on the fecundity and survival and NOT on the transmission dynamics.

Answer: As requested by the reviewer, we have changed the way we phrase our sentence in the part describing our objectives line 153.

Methods

Line 118: From which generation, the authors started the colonization?

Answer: As explained in the manuscript, the colony was started with the help of Pr. Ricardo Molina from eggs from an already existing colonies. Unfortunately, we are unable to answer his question, as our colleague did not provide us with this information. However, according to Pr. Ricardo Molina, the *Ph. perniciosus* colony was established from captures he made in Madrid, Spain, in 1987. The name of this strain is ‘Boadilla *Ph. perniciosus’*.

Line 119: Add the paper of Maroli et al. 2007, Chelbi and Zhioua, 2007, 2019

Answer: As requested by the reviewer, we have added the references line 162.

Line 119: Add a published reference and not submitted (Ref 28 is not acceptable).

Answer: According to PLOS Neglected Tropical Diseases journal guidelines, acceptable references include: published or accepted manuscript and manuscripts on preprint servers, providing the manuscript has a citable DOI or arXiv URL. Reference 28 (now 34, line 163) is accessible on preprint server (BioRxiv, DOI: <https://doi.org/10.1101/2023.07.19.549665>). This reference provides relevant improvements we have made to establish and maintain our sand fly colonies.

Line 132: Please provide the origin of the strains and from which sandfly species it was isolated. Also, add the Gen Bank Accession Number of the complete sequence of the TOSV strain if it has been published in the GenBank database.

Answer: The TOSV virus lineage B was isolated from a human and not from a sand fly. We have added the Genbank ID in our manuscript line 177.

Line 138: Please specify at which passage of viral culture, the TCID50/ml was calculated as well as the name of the method (ref) used in calculation.

Answer: We thank the reviewer for this comment. We used the passage 2 of the viral culture. The information has been added to the manuscript line 185. The name of the method used in calculation is mentioned with the reference later in the Methods, in the specific part on virus detection: “TCID50/ml was calculated according to the Reed-Muench method (33)”.

*Reed LJ, Muench H. A Simple Method of Estimating Fifty Percent Endpoints. Am J Trop Med Hyg. 1938;27(20):493–7.*

Line 149: Add the reference of Maroli et al. 2007.

Answer: If the reviewer talk about this reference “*Maroli, M., Pennisi, M. G., Di Muccio, T., Khoury, C., Gradoni, L., & Gramiccia, M. (2007). Infection of sandflies by a cat naturally infected with Leishmania infantum. Veterinary parasitology, 145(3-4), 357-360*.”, we don’t think this reference is appropriate and linked of this part of the manuscript.

Line 150-151: The authors should report which passages of viral cultures related to these three concentrations

Answer: As requested by the reviewer, we have added this information in our manuscript line 196.

Line 152: Are you using the same generation of Ph. perniciosus? Did the authors examine viral infection in Ph. perniciosus prior to the experiment?

Answer: We thank the reviewer for these questions. As the reviewer is probably aware, it is difficult to realize all the experiments with the same generation of sand flies as we cannot take more than a third of the weekly production (Molina *et al.*, 2017). However, as described in the article, experimental infections were realized according to standard practice for sand flies and as previously described for *Leishmania* infection (Vaselek *et al*., 2020) modified for TOSV. Naturally, our colonies are regularly tested for parasites, bacteria and viruses.

*Molina R, Jiménez MY, Alvar J. Methods in sand fly research. 2017. 108 p.*

*Vaselek, S., Prudhomme, J., Myskova, J., Lestinova, T., Spitzova, T., Bañuls, A. L., & Volf, P. (2020). Comparative study of promastigote-and amastigote-initiated infection of Leishmania infantum (Kinetoplastida: Trypanosomatidae) in* Phlebotomus perniciosus *(Diptera: Psychodidae) conducted in different biosafety level laboratories. Journal of medical entomology, 57(2), 601-607.*

Line 168: What type of measures taken to prevent cross contamination between parts. Salivary glands is the most important organ to be examined after blood digestion.

Answer: To prevent the potential cross contamination between sand fly parts, we performed sterile dissection by cleaning the dissection tools with disinfectant (Anios™ Aniospray Quick Spray Surface Disinfectant, ethanol-based, broad antimicrobial spectrum) and then rinsing with water between each sand fly body part and between each sand fly as well. Regarding the salivary gland examination, we totally agree with the reviewer that these organs are very important to study. In *Phlebotomus* sand flies, salivary glands are in the insect thorax and are extremely tricky to dissect. Here, we were looking at the TOSV infection dynamics and to observe the virus circulation in the vector we separate sand flies in 3 parts: (i) head, (ii) thorax/abdomen and (iii) legs/wings. The presence of virus in wings/legs or head is a proof of systemic infection, the virus disseminates in the secondary tissues. We obtained this relevant information, which is a first step towards understanding TOSV natural cycle, but it would be useful to have more details on TOSV presence in the salivary glands which is the next step.

Line 169: Why including wings and legs? The authors should differentiate between engorged females and females that digested blood. This have to be discussed. Most importantly is to test eggs, larvae, and emerging adults males and females for viral infection.

Answer: We included sand fly wings and legs in order to test for the dissemination of the virus into the haemocoel. Vector competence is a dynamic process that includes at least two successive steps (i) gut infection after an infectious blood meal ingestion (*i.e.* virus has to go through a gut infection barrier), and (ii) virus escape from the gut to reach the haemocoel and secondary organs. Detecting virus in secondary organs like wings and legs allows to estimate dissemination prevalence (*i.e.* the proportion of infected sand flies with a systemic infection). All engorged females were selected post infectious blood meal exposure. They did not digest any blood before the experiment and were sorted 24 hours after the experimental blood meal. All exposed females would eventually digest their infectious blood meal over the course of the experiment. Lastly, we agree with the reviewer that the next step would be to test vertical transmission through eggs, larvae and emerging adults. Our experiments focused on the sand fly life history traits, and we plan to perform the entire cycle test in the future. We added a sentence at the end of the discussion line 645: “Finally, vertical transmission of TOSV was not addressed in this study and further work is required to elucidate the existence and impact of this mode of transmission on the persistence of the virus in the environment and its dispersal”.

Line 172: Please explain the rationale of using only dose 3.

Answer: As vector competence is virus dose-dependent, experimental infection with the highest dose of virus (Dose 3) ensures that a maximum number of sand flies are infected and infectious within a short time after infection. This was done as a proof of concept that we could passively detect the virus in the saliva of the sand flies during sugar feeding.

Line 181: Add the % of hatching.

Answer: As requested by the reviewer, we have added the hatching rate in Table 3 line 431.

Line 184: “adding sand” Please add the reference of Chelbi and Zhioua, 2007.

Answer: As requested by the reviewer, we have added the reference line 231.

Line 190: The cannibalism occurs only when food for larvae is enough. This is not an acceptable reasons. The authors should follow up the life cycle until emergence of adults to be examined for transovarial transmission. The authors should let larvae accomplish their cycle and test the presence of TOSv in eggs, larvae, and emerging adult.

Answer: The larvae were not fed to accurately count them and the eggs. Accurate tracking of sand fly eggs and mobile first instar larvae is meticulous and delicate and can only be achieved under a binocular microscope. In the absence of food, as previously published, cannibalism may occur and bias the results (Srinivasan *et al.* 1992). We agree that it is interesting to assess the likelihood of vertical transmission across virus doses, successive blood meals or incubation times (Manuel *et al.* 2020) to understand the mode of transmission, spread and persistence of this virus in the environment. Nevertheless, isolating the offspring of each female to assess the within-condition variability of this trait and rearing them to adulthood in a Biosafety Level 3 insectary is laborious and time consuming (2 months to complete the life cycle). It was decided not to include this aspect in this work. We considered it to be an important second step, after characterising the effect of virus dose and post-exposure time on vector competence as a first step. Finally, this paper focuses on the effects of infection on the life history traits of infected females. However, in the Discussion line 645, we include a limitation section with a sentence with the following guidance: “Finally, vertical transmission of TOSV was not addressed in this study and further work is required to elucidate the existence and impact of this mode of transmission on the persistence of the virus in the environment and its dispersal”.

*Srinivasan, R., & Panicker, K. N. (1992). Cannibalism among immatures of Phlebotomus papatasi (Diptera: Psychodidae). J. Bombay Nat. Hist. Soc, 89, 386-387.
Manuel, M., Missé, D., & Pompon, J. (2020). Highly efficient vertical transmission for Zika virus in* Aedes aegypti *after long extrinsic incubation time. Pathogens, 9(5), 366.*

Line 195: This is an unacceptable reference (submitted). The authors do not have a long history of sand fly colonization. Reducing the T°C will make the oviposition period longer. Engoged females that do not have access to an egg-laying post will cause a significant stress factors. This must be discussed.

Answer: It is worth noting that we started to work with *Phlebotomus* sand flies with one of the best teams in this domain, Prof. Petr Volf, 7 years ago. Regarding the other comments, we agree that oviposition period is greatly influenced by the temperature. The impact of the presence of the egg-laying pot on stress can indeed be discussed. This section was removed from the manuscript. Here, we just compared females’ survival across blood meal conditions in simple and standardised conditions. The infectious blood meal effect was the main parameter under observation.

Line 197 : Specify these conditions

Answer: As requested by the reviewer, we specified the standard conditions of rearing line 248: “26 ± 1°C, 80% relative humidity (rh), 14h: 10h light: dark cycle”.

Line 202 : It could be interesting to use a lower dose close to natural transmission

Answer: We used the highest dose of virus (dose 3) as proof of concept to test the effect of infection on survival. As a high infection does not impact the sand fly survival, it is unlikely that a lower dose would have an impact on the survival of the sand fly.

Line 233: The authors should define in MM the term infection dynamics. It means to quatify the viral infection in the gut and in hoemocel, then in the salivary galnds. It is a continious process.

Answer: As requested in the reviewer comments above, we added the definition of infection dynamics in the introduction of the manuscript line 106. In sand fly-borne pathogens, the infection dynamic of *Leishmania* parasites is well known but for phleboviruses there is a lack of information, and everything needs to be study. We don’t know if the virus replicates in the salivary glands, in the stomodeal valve (like *Leishmania*) or in another organ of the sand fly. That’s why we first quantify virus in the thorax/abdomen (gut), head and wings/legs (haemocoel) over time to show the systemic infection (the virus circulate and replicate in the vector). Then, the next step will be to quantify the virus in different organs, such as salivary glands or stomodeal valve.

Line 234 : Define in MM systemic infection

Answer: As suggested by the reviewer, we added the definition in brackets line 299: “virus dissemination from sand fly midgut to secondary tissues”.

Results

Line 247: This daily mortality is high. Could you explain the reasons?

Answer: In our sand fly colony, we have a daily natural mortality of approximately 10-20%, which is common in sand fly rearing according to sand fly experts that we have consulted when establishing our colony.

Line 282: Delete “the estimation from 0 to 5”

Answer: As suggested by the reviewer, we deleted this part line 358.

Line 295: 9280 ???

Answer: As explained earlier, we decided to first analyse the samples infected with dose 3, as a proof of concept, to be sure to detect virus within the sand fly parts. The number 9280, calculated according to the TCID50 Reed and Muench 1938 method, is higher at 14 dpi due to virus replication within the sand fly bodies along the time post infection. These details are described in the MM of the manuscript, line 283. However, it would be necessary to replicate this experiment with dose 3 and dose 2.

*Reed LJ, Muench H. A Simple Method of Estimating Fifty Percent Endpoints. Am J Trop Med Hyg. 1938;27(20):493–7*

Line 305-310: It is very important here to clearly clarify the sugar cotton analysis result for the three doses. Did the three batches of females infected with doses 1, 2 and 3 release the virus during sugar feeding.

Answer: In the MM part of the manuscript, line 219, we specified “One cotton per day at two, four, six, eight and 10 days post infection (dpi) with dose 3 only (as a proof of concept, to ensure the viral detection) were analysed with the same RT-qPCR protocol as the sand fly samples”. The sugar cotton analysis was carried out with dose 3 only, as a proof of concept, to ensure the viral detection. Further experiments will be performed on the virus detection in the sugar cotton, with more samples, for dose 1, 2 and 3.

Discussion

Line 360: Please note that microbiota has a great impact of the vector competence.

Answer: We thank the reviewer for this suggestion. In the future, we intend to investigate the impact of microbiota as well on *Ph. perniciosus* vector competence.

Line 366: How you explain the presence of TOSV in wings and legs?

Answer: Heads and wings were separated from the bodies and tested for presence of TOSV to assess whether the virus successfully passed from the midgut to the haemocoel, indicative of virus dissemination throughout the body. As explained earlier, we obviously took every precaution to avoid contaminating the wings and legs when dissecting.

Line 368: Ph. perniciosus as the main vector of TOSV in nature is already confirmed. Thus, the authors only quantified the virus during its replication and dissemination.

Answer: As far we are aware, no study has been published in a scientific journal on TOSV experimental infection dynamics within the vector *Ph. perniciosus*. The vector competence of *Ph. perniciosus* has been confirmed only after field collections in endemic areas. It is therefore important to study the kinetics of virus development in the vector and to show that *Ph. perniciosus* can be infected by TOSV through experimental infections for validation. Experimental infection is one of the key recommendations to validate the vector competence of arthropod species (Wu *et al*. 2022).

*Wu, V. Y., Chen, B., Christofferson, R., Ebel, G., Fagre, A. C., Gallichotte, E. N., ... & Ryan, S. J. (2022). A minimum data standard for vector competence experiments. Scientific Data, 9(1), 634.*

Line 380: The authors should always use the term mosquito-borne arboviruses. It is misleading to use the term arboviruses in the discussion.

Answer: In order not to be misleading, we have replaced the term arboviruses by mosquito-borne viruses, line 477.

Line 387: Please note that the infection related to Ref (12) is through intra-thoracic injection and therefore, you cannot make comparison.

Answer: We completely agree that intra-thoracic injection is different from oral feeding (which is closer to natural conditions of infection). No study has been published in a scientific journal on TOSV experimental infection of the vector *Ph. perniciosus* through an oral blood meal. We are comparing our results with the most similar published experiences. We modified the sentence line 510 as mentioned in the answer below.

Line 391: Please provide other convincing explanation beside the use of different strain.

Answer: As requested by the reviewer on the comment above and this one, we added other explanations for this statement as following: “This discrepancy between the two studies may be due to (i) the utilization of different TOSV lineages or (ii) the experimental infection methods used. Intra-thoracic inoculations of the virus (far from the natural infection route) might increase sand flies mortality rate over time”, lines 510-513 of the manuscript.

Line 392: Not sure, the author should check the dynamics of viral infection in the vector. First it decreases followed by a replication and dessimination.

Answer: We agree that a transient decrease in virus titer can sometimes be observed in arthropod bodies after ingestion of the infectious blood meal. This could represent the transition between the digestion of the infectious blood meal (total virus load decreases with time) and virus production by the infected sand fly. This decrease can only be observed at small timescales. In a global pattern, the virus titre increases over time.

Line 393 : Ref (47) is not approriate.

Answer: As requested by the reviewer, we changed the reference line 515 by the following one: *Lequime, S., Dehecq, J. S., Matheus, S., de Laval, F., Almeras, L., Briolant, S., & Fontaine, A. (2020). Modeling intra-mosquito dynamics of Zika virus and its dose-dependence confirms the low epidemic potential of Aedes albopictus. PLoS Pathogens, 16(12), e1009068.*

Line 405: The authors are assuming that human is a reservoir host for TOSV. This is a miss leading statement and should deleted.

Answer: We agree with the reviewer. We wanted to highlight the implication of a low dose infection. Due to a lack of knowledge, we modify the sentence as following: “Our results showed that TOSV RNA persists in sand flies over 15 days when infected at dose as low as 104 TCID50/ml. This dose infection threshold appears to be compatible with most viral loads found in reservoirs in other arbovirus models. Therefore, sand flies could be infected throughout the viremic period, regardless of whether the host is symptomatic”, lines 523-541 of the manuscript.

Line 408 : “female sand flies die before taking a second blood meal”. This is totally wrong. Ref 28 should be replaced. Again, the authors do not have good experience in sand fly biology.

Answer: We modified the sentence which wasn’t clear: “In colony settings, the blood-fed females died most of the time after egg-laying, before taking a second blood meal (29), precluding to measure transmission during a second artificial blood meal” and we changed the reference lines 544-546. We, of course, know the sand fly biology and that in nature, female take several blood meals during their life. However, in the laboratory, it is known, especially for *Phlebotomus* genus, that must of the females die after laying eggs in colony as already widely published. *E.g*. “Identify females who have done oviposition that in most cases will have died” (Molina *et al*., 2017) or “After laying the eggs, females die or could be killed for species identification” (Volf and Volfora, 2011).

Line 424: The authors should demonstrate infection during sugar co-feeding.

Answer: The next step of this study is to demonstrate the infection during sugar co-feeding for *Ph. perniciosus* and other species, as mentioned in our discussion lines 562-570: “To better understand the transmission dynamics and be able to predict TOSV emergence it would be crucial to investigate further the possible alternative transmission routes of TOSV between sand flies through their sugar meal”.

Line 427: The authors emphasize on the viral transmission through sugar feeding and making assumption with similarity for blood feeding viral transmission

Answer: Indeed, we are opening the discussion on possible alternative transmission routes of TOSV between sand flies. See discussion part of the manuscript, line 641.

Most important concerns in the discussion

The authors should compare and discuss the difference between intrathoracic inoculation and membrane feeding. Sand flies are refractory to oral infection. Phlebovirus are maintained in nature through transovarial transmission. Please refer to Tesh and Modi, Am J Trop Med and Hyg 1984 33: 1007-1016.

Answer: We do not agree with the reviewer saying that sand flies are refractory to the oral infection. After the mentioned study by Tesh and Modi in 1984, some authors published the evidence of sand fly infection by other sandfly-borne virus (*e.g.* Massilia virus) through oral experimental infection (Jancarova *et al*., 2019), which is the closest experimental infection to natural infection compared to intra-thoracic inoculation. Moreover, refractoriness to oral feeding may be due to the technique used by the experimenter in Tesh and Modi in 1984. Here, we were able to infect our sand flies by oral feeding, which highlight the feasibility of this transmission route.

In addition, the authors should provide convincing proof that wigs and legs were not infect through sugar feeding.

Answer: As the wings and legs were tested at different time post infectious blood feeding, the fact that wings and legs become infected cannot be associated with sugar feeding.

Why the authors exclude dogs from the study? This is an important missing data.

Answer: It is true that it is important to complete the entire cycle including the host. Nevertheless, before performing experiments on dogs (experimentations on such animals is another story), our hypothesis requires strong proofs and sand flies (although complicated) are good models to understand better the biological cycle of TOSV. Furthermore, we do not know yet with certainty if dog is a reservoir or an epidemiological dead end.

48 hrs after blood feeding, engorged sandflies were stored in 70% alcohol for further DNA extraction and qPCR for *L. donovani*. The normal cycle of *L. donovani* within the sandfly takes 8-10 days to reach stationary phase. Why the authors did not allow for a full cycle accomplishment of *L. donovani* within the sandfly.

Answer: The authors do not understand the reviewer's question. Our manuscript describes the Toscana virus infection dynamics within its main vector, we have not worked with the parasite *L. donovani*.

Results

Since xenodiagnosis was repeated throughout the year, the authors should report results at each time.

Answer: The authors do not understand this comment. We never practiced xenodiagnosis in our study.

Discussion

The authors should report studies concerning xenodiagnoses performed on humans.

Taking into account that dogs are considered as reservoir hosts for *L. donovani* (Jumbulingam et al., 2017), the authors should discuss the rationale of not testing dogs.

Answer: The authors do not understand the reviewer's comment. Our manuscript describes the Toscana virus infection dynamics within its main vector, we have not worked with the parasite *L. donovani*, and we did not perform xenodiagnosis.

**Reviewer #3**

Methods

A standard curve was carried out for absolute quantification of TOSV RNA in sugar soaked cotton pads through RT-qPCR to obtain the virus detection limit > Was it also done for samples? Or only cottons?

Answer: We apologize for the lack of clarity. Viral genomic titers were quantified using a standard curve for all samples (Figure 3). We modified the sentence line 275 “A standard curve was carried out for absolute quantification of TOSV RNA (**S1 Appendix**).”

samples with Ct value < 40 were considered positive > why not using standard curve detection limit value?

Answer: We clarified this point in the manuscript. A Ct of 40 corresponded to less than 10^-4^ copies/ml of viral RNA based on our system. We considered as negative all Ct value > to 36. The virus detection limit is set at 10^-2^ RNA copies/ml.

Was TOSV values normalised on sandfly standard gene? To describe.

Answer: In our experiment we didn’t normalise the TOSV RNA copy number on sand fly standard gene since we have a standardised protocol with aiming to identify the viral particle number per body part, safely assuming that the number of cells per body parts are equivalent across flies. This is commonly done in other mosquito artificial infection articles (Gabiane *et al*., 2024). We added this information in the manuscript lines 276-278.

*Gabiane, et al., 2024. Evaluating vector competence for Yellow fever in the Caribbean. Nature Communications, 15(1), p.1236.*

To prevent the fungi development which could affect the survival of the sand flies, dead females were removed > how was it performed. I am just concerned if using anesthesia by cold, groups with dead sandflies were therefore more stressed thn other groups so this could biais the outcome on survival.

Answer: For the survival analysis, we anesthetised sand flies by ice in each group, control and test, to avoid this bias but only once (after the blood feeding, to transfer engorged flies in pot). In our BSL-3 facility, we follow a strict protocol with specific secured pots in which we place the sand flies for the follow-up experiment (see picture below). It is possible to remove the tiny dead sand flies through the ‘door’ of this pot, avoiding the risk of escaping flies. This is how we removed the dead individuals during the 50-day survival follow-up assay. It is very important to avoid fungi development for sand flies.

Between 10 to 30 individuals were dissected in three parts: head, body (thorax and abdomen), wings and legs, every day for 15 days > It is mentioned L246 that sand flies were dissected every two days. Please correct.

Answer: As requested by the reviewer, we corrected this part in our manuscript line 217.

Results

Figure 3. Please do a graph for dose 1. Viral loads are important but on your qPCR graph this is bars so we do not have any idea of the prevalence but just a mean of the whole. Please do a box plot showing all individual values, with qPCR detection limit shown on the graph (dotted line?) and prevalence in % and total number of sandlflies tested visible in figure.

Answer: We modified the Figure 3 following the reviewer recommendations line 338. The authors think the graph for dose 1 (10^2^ TCID50/ml) is not necessary to show in the manuscript, as the virus didn’t disseminate in infected sand flies (no positive heads or wings, legs) and the numbers of dissected individuals were too low (see table and Figure below).

**Table**. Raw data for infection dose 1

Mean TOSV RNA copies/ml (log10)

| Days post infection with dose 1 | Body | Head | Wings Legs |
| --- | --- | --- | --- |
| 3 | 12647.2 | 0 | 0 |
| 5 | 0 | 0 | 0 |
| 7 | 670.729 | 0 | 0 |
| 9 | 0 | 0 | 0 |
| 11 | 0 | 0 | 0 |
| 13 | 0 | 0 | 0 |
| 15 | 0 | 0 | 0 |

**Figure**. Mean log10 viral particles in body, head, wings and legs of infected female sand flies fed with dose 1 (10^2^ TCID_50_/ml) of Toscana virus over time post infection. Toscana virus RNA levels in the sand fly parts were quantified by RTqPCR. The error bars correspond to the standard deviation.

Why using different colours between dose 2 and 3 on same figure but different panels eg bodies is yellow and then red. Please be homogenous for the ease of readers. Be mindful of colourblind people.

Legends are a bit short, please add more details, method, numbers, colour code, what the error bars or box plot lines correspond to (sem or sed?) etc. Please refer to published papers to see what a legend should show.

Answer: We modified the Figure 3 and its legend following the reviewer recommendations line 347. The number of dissected sand flies for each batch is represented in Table 1. We have chosen the colour yellow to represent infection dose 2 and red for infection dose 3, to be homogenous with our life history traits experiment where the boxplot is yellow for dose 2 and red for dose 3. According to the reviewer suggestions, we changed the Figure 3 colours.

Increase in viral loads at dose 2. Statistics should be there to support this statement. How were normalised the viral loads, any standard gene?

Answer: As previously explained, in our experiment we didn’t normalise the TOSV RNA copy number on sand fly standard gene. We made the safe assumption that the number of cells across body parts from several sand flies is similar and that virus titer can be directly compared across conditions. This is a common practice as described in Gabiane *et al*., 2024, Lequime *et al.*, 2020 and Bellone *et al.*, 2020.

*Lequime, S., Dehecq, J. S., Matheus, S., de Laval, F., Almeras, L., Briolant, S., & Fontaine, A. (2020). Modeling intra-mosquito dynamics of Zika virus and its dose-dependence confirms the low epidemic potential of* Aedes albopictus*. PLoS Pathogens, 16(12), e1009068.*

*Bellone, R., Lequime, S., Jupille, H., Göertz, G. P., Aubry, F., Mousson, L., ... & Failloux, A. B. (2020). Experimental adaptation of dengue virus 1 to* Aedes albopictus *mosquitoes by in vivo selection. Scientific reports, 10(1), 18404.*

In sand flies infected with dose 2, up to 21 days is not shown. Either you do not speak about it or you show it, the number of sandflies at these days is enough according to me, but indeed they come from only one replicate. Maybe best to leave the description of these day out.

Answer: As suggested by the reviewer, we deleted the information about sand flies infected up to 15 days in our manuscript line 509.

L266 to 270: The analysis of significance of the TOSV dose on post-infection should be shown (table with comparisons and stat). The ext should be more dtailed, which times, which tissue, for instance I can t see any effect of the dose in bodies. The text should be clearer to well describe what s the effect of what and what is significant or not.

Answer: We modified this part of our manuscript. As explained in the Methods, the significance of the TOSV doses (10^4^ and 10^6^ TCID50/ml) and the effect of time post-infection on the infection dynamics was tested by general linear mixed models (GLM). The authors think it is not appropriate to show all the details about the statistical analysis, but important to mention the p-value. However, we share it below for the reviewer and we can share it in Supplementary data if needed.

Glm body~dose

Family: binomial ( logit )

Formula: Body ~ factor(Dose) + (1 | Dpi)

AIC	BIC	logLik deviance df.resid

183.8	194.2	-88.9	177.8	233

Random effects:

Conditional model:

Groups Name	Variance Std.Dev.

Dpi	(Intercept) 2.555e-09 5.055e-05

Number of obs: 236, groups: Dpi, 7

Conditional model:

Estimate Std. Error z value Pr(>|z|)

(Intercept)	1.1811	0.2053	5.752 8.8e-09 ***

factor(Dose)6	2.0377	0.5497	3.707 0.00021 ***

Table 2 should indicate that the values unit is 10 to the 6 (not only in legend). There si a mistake I guess for day 14.

Answer: The values in this table are in TCID50/ml (not 10 to the 6), we wanted to show that the samples were infectious by using the titration method described by Reed and Muench 1938. We decided to first analyse the samples infected with dose 3, as a proof of concept, to be sure to detect virus within the sand fly parts. The number 9280 (at 14dpi) is higher at 14 dpi due to virus replication within the sand fly bodies along the time post infection. These details are described in the MM of the manuscript, line 283. As explained in the discussion line 641, we need to replicate this experiment.

*Reed LJ, Muench H. A Simple Method of Estimating Fifty Percent Endpoints. Am J Trop Med Hyg. 1938;27(20):493–7*

Virus infectivity in sand fly samples: It would be better if there was a figure (in supplementary?) showing on the same graph (two Y axis) both the qPCR and titration data for same samples (the 84 samples) so we can appreciate if viral loads by qPCR is indeed a valid method. And not just a table 2 with only a mean of titres. While qPCR can be a good method to asses viral loads, this needs to be confirmed, especially at the light that none of the legs and heads samples were positive by titration while virus detected by qPCR.

Answer: The TOSV titers presented in Table 2 are not mean titers, they represent the seven titers measured in the seven positive sand fly bodies (thorax/abdomen), over the 84 samples analysed, after titration using the TCID_50_ method. As we had only 7 positive samples for this experiment, we decided to represent the data in a Table instead of a figure. The important result of this pilot study is that sand fly body stay infectious up to 14 days. As mentioned in the text line 641, these experiments will be repeated, and futures data will be represented as suggested by the reviewer.

L309: viral particles were detected > no, RNA was detected.

Answer: We corrected the sentence in our manuscript line 394.

A figure should accompany data described L305 to 310.

Answer: This experiment aimed at detecting the virus in sugar cottons. We only had 3 data points: virus RNA copies at 2 (n=30 sand fly pool), 6 (n=27 sand fly pool) and 8 (n=20 sand fly pool) days post infection. We decided that a figure for three points was not relevant. As mentioned in the text line 641, these experiments will be repeated.

Infection impact on vector fecundity life history traits > please show a table with all statistics even if not significant.

Answer: We added the following Table, with all descriptive statistics, in supporting information.

**S3 Appendix**. Descriptive statistics for the analysis of the infection impact on sand fly life history traits infected by dose 2 (10^4^ TCID_50_/ml) and dose 3 (10^6^ TCID_50_/ml)

| **Group** | **Glm statistical analysis** | | |
| --- | --- | --- | --- |
|  | *(Signif. codes: 0.0001 ‘***’ 0.001 ‘**’ 0.01 ‘*’ 0.05 ‘.’ 0.1 ‘ ’ 1)* | | |
|  | *Std. Error* | *t value* | *Pr(>\|t\|)* |
|  | **Survival time** | | |
| Dose 2 | 0.5263 | 2.970 | 0.0541 |
| Dose 3 | 0.5263 | 1.836 | 0.06810 |
|  | **No. eggs** | | |
| Dose 2 | 3.0676 | 1.107 | 0.270 |
| Dose 3 | 3.0926 | -0.284 | 0.777 |
|  | **Oviposition time** | | |
| Dose 2 | 4.836 | -2.070 | 0.0605 |
| Dose 3 | 4.836 | -0.956 | 0.3412 |
|  | **No. larvae** | | |
| Dose 2 | 2.6273 | -0.255 | 0.799 |
| Dose 3 | 2.6273 | -0.843 | 0.401 |
|  | **Hatching time** | | |
| Dose 2 | 0.2311 | 2.274 | 0.0247 * |
| Dose 3 | 0.2311 | 5.284 | 5.61e-07 *** |

only hatching time of test groups dose 2 (GLM test, P = 0.0247) and dose 3 (GLM test, P = 5.61e-07) > does this correspond to p value on figure 5? If yes, the figure should be cited here.

Answer: Yes, these data correspond to p-values on Fig 5. We added this information in our manuscript line 422.

Conclusions

Authors mention in the abstract that this research confirms vector competence. However, dissemination is not a proof of vector competence. I d recommend being more cautious and say something like potential vector competence. Or change systemic dissemination to presence of virus RNA in saliva/cotton…

Answer: We changed the sentence to nuance our talk regarding the potential vector competence as recommended, but we kept systemic dissemination since the data clearly show it.

L57: on vector hatching time leading to a delayed emergence of infected sand flies > check wording here so this is correct. Larval hatching and delayed emergence of corresponding adults (not infected but their mothers were), modify the sentence. Actually in methods it is mentioned that alrvae were killed so what is delayed emergence? Eggs hatched into larvae and adults emerge from pupae. Use the correct vocabulary to avoid any confusion.

Answer: We changed the sentence in the author summary to avoid any confusion: “Our study reveals an impact of TOSV infection on vector egg hatching time that could lead to a delayed emergence of infected sand flies, with a potential impact on transmission”.

As mentioned in general comments, while it is not highly problematic to use qPCR, conclusions and discussion must stick to the data and authors should not overstate. The results as they are interesting and constitute a good advance in the field of sandfly vector competence.

Maybe try to make the point that finding RNA copies in head or in cotton means that there has been dissemination or presence of virus in salivary glands (RNA of dead particles could be spitted?) but stay cautious as it does not mean it is still infectious or that the saliva was. Just stay on the cautious side and highlight the limits of the study.

Answer: We changed some of our sentences in the discussion to moderate our statements and we added a part in the discussion (line 641) with the limits of the study.

The authors should carefully revise their manuscript, so their conclusions fit to their data and not to overstate. The limitations of RTqPCR should be highlighted and discussed (see figure suggested to compare qPCR and titrations on samples measured both ways)

Answer: We thank the reviewer for these suggestions. We have revised our manuscript to avoid overstatements, but it is rather difficult to add the titration results in a figure due to the small effective number. For this reason, we have chosen to present the data in a Table 2.

Examples where authors should revise (not exhaustive, please carefully check all statements in discussion as I do not list them all).

“we found viral particles in head and thorax, where the salivary glands are presents (42), until at least 15 dpi, as well as in wings and legs (Fig 3)”. On fig 3 these are RNA not viral particles.

“virus particles did not disseminate in the vector, and only a few body samples were tested positive before sand fly defecation”

“TOSV disseminated into the secondary tissues and the infection persisted for at least 15 days (Fig 4)”, only tested for dose 3 (infection means infectious virus particles, not RNA which can be traces of dead particles).

L 402, L423 (sugar transmission, watch as you show RNA here) Etc

Answer: We corrected some of our sentences and changed words in the discussion to avoid overstatement.

L460 to463: it is a bit of a stretch here. Transovarial transmission does not mean necessarily adult females infected. You also never show that you have transovarial transmission in your model. There is also a lack of clarity between hatching and emergence. They never tested emergence, so stick to hatching. Rather than over hypothesising on adult mergence, I d rather try to discuss why hatching is longer, what would be the underlying mechanism (reproductive process less effective in inf females, leading to less reserves?)

Answer: We modified text in the manuscript line 612 to clarify the part about egg hatching. According to the only one study (Tesh and Modi, 1987) showing TOSV transovarial transmission in *Ph. perniciosus*, the transmission rate to the next adult generation F1 is up to 90%, which is considerable. They also showed the TOSV vertical transmission for 13 consecutive sand fly generations. However, it was not our objective to show the TOSV transovarial transmission here, even if it will be interesting to further study it. We didn’t follow the development of sand fly until adult emergence in this pilot study because it’s a 2 month daily follow up in confined BSL-3 facilities; and we must first write precise protocols and have them validated by the BSL-3 manager before starting this long experiment. It will be the next step in our study to better understand TOSV natural cycle.

*Tesh, R. B., and Modi. G. B. "Maintenance of Toscana virus in* Phlebotomus perniciosus *by vertical transmission." The American journal of tropical medicine and hygiene 36.1 (1987): 189-193.*

I am not too sure we can compare EIP in sandflies and mosquitoes. In addition, some arboviruses transmitted by mosquitoes have a very short EIP eg CHIKV.

Answer: We agree with this statement. Here, we demonstrated a link between the dose of virus and the time to systemic infection, which can be considered as a proxy to EIP. EIP distributions are difficult to compare across studies and models. We modified this paragraph in our manuscript line 633.

The discussion is generally a bit too long, but I guess this can be shortened by removing all overstatement and focusing on the data without stretching on hypotheses.

Answer: We shortened the discuss as much as possible.

Editorial and Data Presentation Modifications?

It would be good to mention in introduction, if known, the approx. numbers or %age of meningitis and encephalitis.

Answer: We thank the reviewer for this comment, we added information into the introduction line 88: “A total of 73.4% of patients suffering from meningitis and encephalitis present at least one of neurological manifestation due to TOSV infection (under review)”. We mentioned ‘under revision’ because one of the co-authors, Dr. Nazli Ayhan, has just submitted this TOSV literature review which has not yet been published.

L32 : the virus's > the viral

Answer: We corrected it in our manuscript line 36.

L34: vectorial competence and capacity > vector competence and vectorial capacity

Answer: We corrected it in our manuscript line 38.

L37: though> through

Answer: We corrected it in our manuscript line 41.

L38: potentially resulting in a shorter extrinsic incubation period > shorter than what?

Answer: We corrected it in our manuscript with “short” instead of “shorter” line 43.

L41: TOSV’s > TOSV. In general, check all the possessive forms as this is overused at different places in the manuscript

Answer: We corrected it in our manuscript line 46 and all over the manuscript.

L63: space between tri and segmented to remove

Answer: We corrected it in our manuscript line 78.

L138: of 0.1 after five days of post infection> and supernatant collected after five days? Clarification of supernatant?

Answer: Indeed, the supernatant is collected, aliquoted and stored in -80°C. We clarified the text line 184.

L144: infection> infections

Answer: We corrected it in our manuscript line 190.

The three doses are called sometimes small, medium and large dose, and sometimes 1,2,3. Find one notation and keep it through the manuscript. Actually it would be easier to write the dose each time (text and figure legends) instead of 1, 2, 3 or small etc

Answer: We changed small, medium and large for dose 1, 2 and 3 in our manuscript line 198.

L181: the traits most important in the virus transmission > other traits are important so rephrase eg vector survival and fecundity, two important traits…

Answer: We corrected this phrase in the Method part of the manuscript line 228.

L287: tree > three

Answer: We corrected it in our manuscript line 362.

L324: eggs and larvae numbers, oviposition and hatching times> please invert so respect the chronology

Answer: We corrected it in our manuscript line 409.

L336: standard error > sem or sd?

Answer: We corrected in the manuscript line 431; it is standard deviation (sd).

L344: p-value significance: 0 '***' > something missing here, cannot be 0 but 0.0001?

Answer: We corrected as 0.0001 ‘***’ in the title of Fig 5 line 442.

L354: Vectorial competence > vector competence and vectorial capacity . Please check all the manuscript

Answer: We corrected it along the manuscript.

L361: vector competence is the ability to be infected and further transmit ( not just transmit)

Answer: We corrected it in our manuscript line 458.

L 364: where it may be released in saliva during a subsequent blood meal > released in saliva and transmitted during a subsequent blood meal

Answer: We corrected it in our manuscript line 461.

L 373: only a few body samples were tested positive before sand fly defecation> can you clarify why mentioning defecation here? What s the significance? Btw, prevalence must be shown for such statements.

Answer: We mentioned the defecation here, to precise that sand flies digested and excreted the blood meal, meaning that TOSV RNA found in these samples could come from the un-excreted blood bolus still present in the sand fly midgut. We added a clarification in the manuscript line 470.

L380: with other arbovirus model> with another… or with …models

Answer: We corrected it in our manuscript line 477.

L384: are generally low and transient, often necessitating the ingestion of a substantial viral load by the vectors > not sure what is the causal link here, not clear. Please reformulate.

Answer: We agree with the reviewer, there is no causal link. We have therefore modified and separated it into two sentences line 504.

L408: female sand flies die before taking a second blood meal > I guess this depends when you offer them a second blood meal.. please clarify, is this that they die before saliva is infectious??? It does not makes sense as you manage to get RNA on cottons from still alive females.

Answer: We corrected it in our manuscript line 544.

L460: As the transovarial transmission of TOSV exists in Ph. Perniciosus > add ref

Answer: The reference is already at the end of the sentence line 614.

Summary and General Comments

In this study, Laroche at al. analyse TOSV infection dynamics in the sandfly Phlebotomus perniciosus, a main vector of TOSV. They also analyse the impact of infection on different fitness traits of the vector, survival, fertility and fecundity. This is quite an impressive study, considering the high complexity of working and breeding sandflies, with replicates and high number of flies. Although some conclusions can be made around infectivity, dissemination and transmission, most of the data were obtained by qPCR which does not measure infectious viral particles. While I appreciate the complexity of such experiments in a BSL3 lab, and that the results are valid experimentally, I think that some conclusions are a bit overstated, ie a RNA copy does not prove infectivity. Saying this, data obtained by qPCR are still very interesting and authors can pull some conclusions from it but they should revise the manuscript not to overstate and highlight the limitations. See my other comments in other sections about this.

Overall, the manuscript is well written, methods and results well described. I suggested some minor modifications and some changes in result presentation, including a couple more figures and more description in legends.

Considering that this study is one of the very few in this field (sandflies and arboviruses), this manuscript is really of interest and merits publication in Plos NTD. However, the authors should carefully revise their manuscript according to my comments to improve its quality and that readers are aware of some limitations. I clicked major revision, but they are all text edits.

Answer: We thank the reviewer for her/his valuable comments. The reviewer might be right about being a bit overstated however our results are not only based on PCR results, but also confirmed by TCID_50_ assay for several samples which is a solid proof of concept. Anyway, we tried to improve the text in limiting the overstatement of the data. These data are essential to further our understanding of TOSV infection dynamics in the vector and open up new avenues of research for future experiments.
